# An *ADAMTS3* missense variant is associated with Norwich Terrier upper airway syndrome

**DOI:** 10.1371/journal.pgen.1008102

**Published:** 2019-05-16

**Authors:** Thomas W. Marchant, Elisabeth Dietschi, Ulrich Rytz, Peter Schawalder, Vidhya Jagannathan, Sheida Hadji Rasouliha, Corinne Gurtner, Andreas S. Waldvogel, Ronan S. Harrington, Michaela Drögemüller, Jeffrey Kidd, Elaine A. Ostrander, Amanda Warr, Mick Watson, David Argyle, Gert Ter Haar, Dylan N. Clements, Tosso Leeb, Jeffrey J. Schoenebeck

**Affiliations:** 1 The Roslin Institute and Royal (Dick) School for Veterinary Studies, University of Edinburgh, Easter Bush, Midlothian, United Kingdom; 2 Institute of Genetics, Vetsuisse Faculty, University of Bern, Bern, Switzerland; 3 Department of Clinical Veterinary Medicine, Division of Small Animal Surgery, Vetsuisse Faculty, University of Bern, Bern, Switzerland; 4 Institute for Animal Pathology, Department of Infectious Diseases and Pathobiology, Vetsuisse Faculty, University of Bern, Bern, Switzerland; 5 Department of Human Genetics, University of Michigan Medical School, Ann Arbor, Michigan, United States of America; 6 Cancer Genetics and Comparative Genomics Branch, National Human Genome Research Institute, National Institutes of Health, Bethesda, Maryland, United States of America; 7 Department of Clinical Sciences and Services, Royal Veterinary College, Hertfordshire, United Kingdom; Stanford University School of Medicine, UNITED STATES

## Abstract

In flat-faced dog breeds, air resistance caused by skull conformation is believed to be a major determinant of Brachycephalic Obstructive Airway Syndrome (BOAS). The clinical presentation of BOAS is heterogeneous, suggesting determinants independent of skull conformation contribute to airway disease. Norwich Terriers, a mesocephalic breed, are predisposed to Upper Airway Syndrome (UAS), a disease whose pathological features overlap with BOAS. Our health screening clinic examined and scored the airways of 401 Norwich terriers by laryngoscopy. Genome-wide association analyses of UAS-related pathologies revealed a genetic association on canine chromosome 13 (rs9043975, *p* = 7.79x10^-16^). Whole genome resequencing was used to identify causal variant(s) within a 414 kb critical interval. This approach highlighted an error in the CanFam3.1 dog assembly, which when resolved, led to the discovery of a c.2786G>A missense variant in exon 20 of the positional candidate gene, ADAM metallopeptidase with thrombospondin type 1 motif 3 (*ADAMTS3*). In addition to segregating with UAS amongst Norwich Terriers, the *ADAMTS3* c.2786G>A risk allele frequency was enriched among the BOAS-susceptible French and (English) Bulldogs. Previous studies indicate that ADAMTS3 loss of function results in lymphoedema. Our results suggest a new paradigm in the understanding of canine upper airway disease aetiology: airway oedema caused by disruption of *ADAMTS3* predisposes dogs to respiratory obstruction. These findings will enhance breeding practices and could refine the prognostics of surgical interventions that are often used to treat airway obstruction.

## Author summary

Respiratory diseases are prevalent across dog breeds, particularly in brachycephalic breeds such as the Bulldog and French bulldog. The flat facial conformation of these breeds has long been assumed to be the major predisposing factor, however, the underlying genetics of their respiratory condition has never been elucidated. We became interested in the Norwich Terrier, a breed presenting with many of the same respiratory disease symptoms as the Bulldog. A distinction, however, is that the Norwich terrier is not considered to be a brachycephalic breed and so presented an opportunity to dissociate respiratory disease from head conformation. We performed a genome-wide association analysis for respiratory disease severity in the Norwich Terrier and resolved an association on chromosome 13 to a missense mutation in *ADAMTS3*. Variants in this gene were previously shown to cause an oedematous phenotype–a disease characteristic in the airways of affected Norwich Terriers and brachycephalic dogs alike. We screened over 100 breeds for the *ADAMTS3* variant and found that it is enriched in the Norwich Terrier, Bulldog and French Bulldog. This discovery changes how we view respiratory disease predisposition in the dog, offers potential genetic screens and highlights a new biological function for *ADAMTS3*.

## Introduction

Amongst dogs, brachycephaly describes the head conformation of many popular breeds including the Bulldog, French Bulldog and Pug. This trait is grossly characterised by the concurrent rostrocaudal shortening and mediolateral widening of the skull and is accompanied by skin folds of the face. The structural discordance between the reduced facial skeleton and its overlying soft tissues such as the wrinkled skin folds underpins these breeds’ iconic looks, but these artificially selected aesthetics are under increasing scrutiny for their association with health problems including breathing difficulties.

It is thought that soft tissues of the upper respiratory tract such as the nostrils, nasal mucosa of the turbinates and soft palate do not scale proportionately with reductions in the midface skeleton [1]. Misconfiguration of respiratory soft tissue restricts airflow and increases negative pressure within the airway [1,2]. This predisposes brachycephalic dogs to Brachycephalic Obstructive Airway Syndrome (BOAS). Dogs diagnosed with BOAS can have stenotic nares, elongated soft palates and oversized, caudally protruding nasal turbinates [2–7]. Airway resistance caused by these tissue anomalies is believed to induce pathological remodelling of additional tissues including tonsil and laryngeal saccule eversion, oedema of the nasopharynx, laryngeal collapse, tracheal hypoplasia and exacerbation of the thickening and elongation of the soft palate [2,8,9]. Collectively, these perturbations severely impact the wellbeing of affected individuals by increasing their respiratory effort, resulting in laboured breathing, intolerance to heat/exercise, cyanosis and collapse [6,7].

The clinical assessment of the respiratory obstruction is often based on the grading of clinical symptoms, diagnostic imaging, and more recently, whole-body barometric plethysmography [6,10,11]. Treatment options for BOAS include anti-inflammatory medication which can reduce swelling/oedema acutely, however corrective surgery is often required to alleviate the condition [12]. Rhinoplasty of the nares, excision of the caudal aspect of the soft palate and aberrant turbinates, removal of the laryngeal saccules and tonsillectomy are the most common surgical procedures, which generally have mixed prognoses [2,3,6,12–14]. The number of patients requiring surgical treatment is expected to rise notably with the rapid increase in popularity of brachycephalic breeds. The costs and morbidity of surgical treatment are a welfare concern for both owners and their dogs, with complications reported in up to 25% and mortality in as many as 5% of cases treated surgically [14].

We and others have studied the underlying genetics of canine skull shape variation [15–19]. Variants in the *BMP3* and *SMOC2* genes are associated with canine brachycephaly, however the contribution of these variants to BOAS pathogenesis is unclear. Moreover, variants in both of these genes appear largely fixed among brachycephalic breeds that are at greatest risk of developing BOAS and yet the incidence and severity of BOAS differs between them [11,20]. BOAS heterogeneity may also be influenced by environmental and epigenetic factors, as well as other genetic modifiers segregating among dog populations. Under this premise, we became interested in the presentation of a respiratory condition remarkably similar to BOAS which has been identified in Norwich Terriers.

As their name suggests, Norwich Terriers originate from south eastern UK where they were used for rodent control. Today, Norwich Terriers are recognised by all major kennel clubs and are known for their short, stocky build and prick ears. Dietschi *et al*. first described the presentation of Upper Airway Syndrome (UAS) in the Norwich Terrier. Although they are not considered a brachycephalic breed, affected Norwich Terriers present many of the hallmarks of BOAS including elongated and thickened soft palates, oedema of the nasopharynx and everted laryngeal saccules [21,22]. The closely related Norfolk Terrier, a breed that officially split from the Norwich Terrier in 1964, is seemingly unaffected by UAS, suggesting genetic predisposition in the Norwich Terriers.

Moreover, anecdotes from breeders regarding more recent dog generations, suggested that some Norwich Terriers appeared shorter-faced than those from earlier generations (personal communication to JS). Indeed, Koch *et al*. postulated that selective breeding is driving Norwich Terriers to become brachycephalic [9,23,24]. Spurred on by these observations, and the possibility of uncovering genetic modifiers that increase respiratory obstruction risk, we sought to understand the genetic basis of Norwich Terrier UAS.

## Results

### Skull morphology of the Norwich Terrier

**Fig 1:**
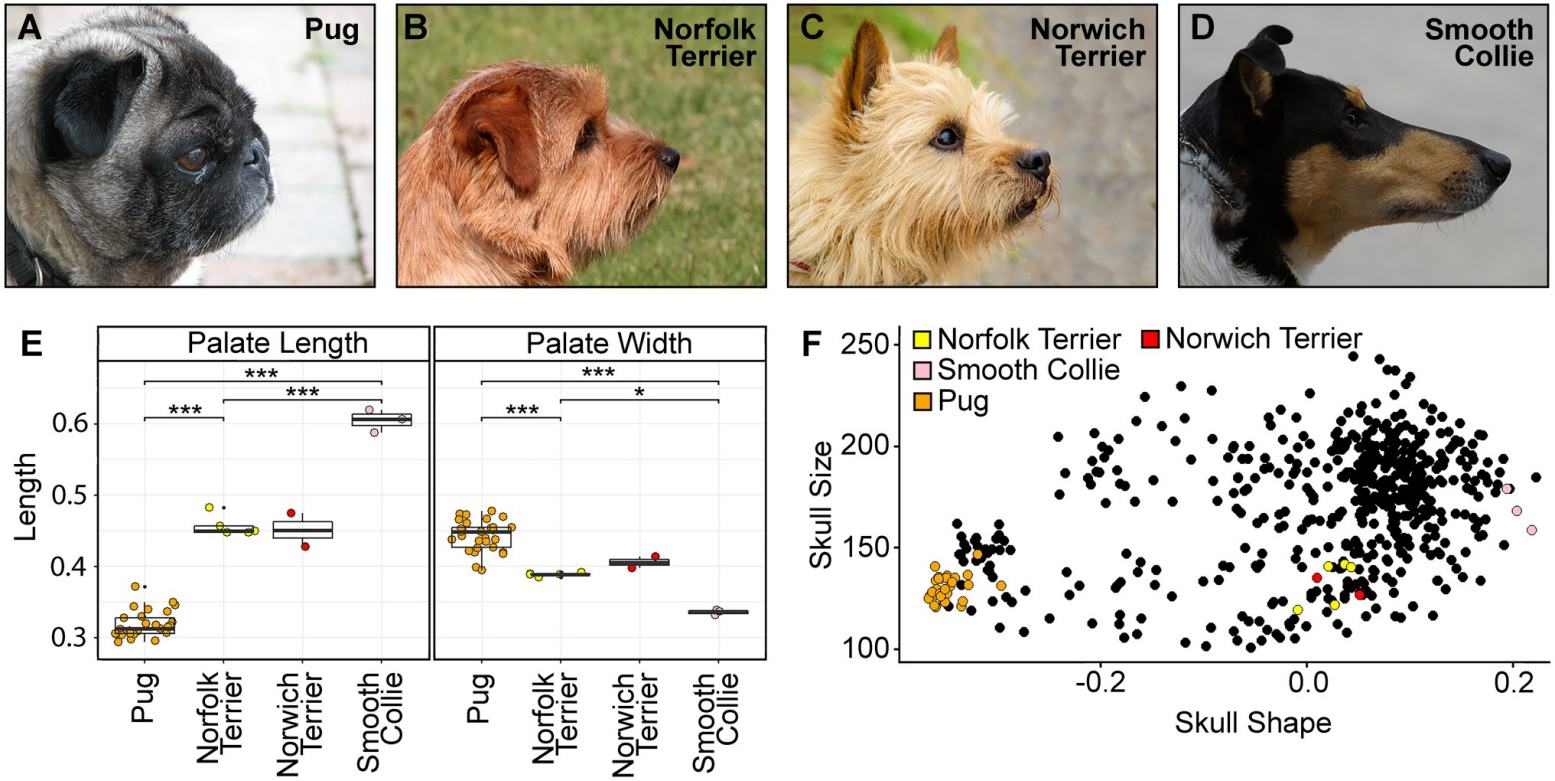
Morphology and respiratory distress. Lateral head profiles of the (A) brachycephalic Pug, (B) mesocephalic Norfolk Terrier, (C) mesocephalic Norwich Terrier and (D) dolichocephalic Smooth Collie. Images are not to scale. (E) Measurements of the canine hard palate normalised for skull size indicate that the rostrum shape of the Norwich Terrier and Norfolk Terrier is intermediate to that of extreme brachycephalic (Pug) and dolichocephalic (Smooth Collie) dogs. Mann-Whitney-Wilcoxon and Kolmogorov-Smirnov tests <0.05 *; <0.01 **; <0.001 ***. (F) Geometric morphometric analysis of 96 domestic dog breeds reveals that the Norwich Terrier and Norfolk Terrier occupy a distinct morphospace from the brachycephalic breeds such as the Pug, the latter which is susceptible to BOAS. Skull size is scored by neurocranium centroid size (y-axis) and skull shape is scored by viscerocranium PC1 (x-axis). Photo credits: Matthew Carr (Pug), Anne Johnsen (Norfolk Terrier), Dan Kyprianou (Norwich Terrier), Bev White (Smooth Collie).

There is a continuum of head shapes observed across the domestic dog population ranging from the extreme brachycephalic to dolichocephalic conformations as represented by the profiles of the Pug and Smooth Collie, respectively (Fig 1A and 1D). Respiratory tract disorders are markedly enriched amongst brachycephalic breeds such as the Pug, Bulldog, French Bulldog, Shih Tzu as well as the Norwich Terrier. The latter is not considered to be a brachycephalic breed, nor is the Norfolk Terrier (Fig 1B and 1C). Rather both are generally considered “mesocephalic”. Indeed, linear measurements of the Norwich Terrier hard palate revealed intermediate palate dimensions between the extremes of facial morphology represented by the Pug and Smooth Collie (Fig 1E). Furthermore, geometric morphometric analysis of the canine rostrum revealed that the Norwich Terrier occupies a morphospace distinct from classic brachycephalic breeds such as the Pug (Fig 1F). For both linear measurements and geometric morphometrics, our data do not indicate a gross morphological difference between the Norwich Terrier and Norfolk Terrier.

### Norwich Terrier upper airway assessment

**Fig 2:**
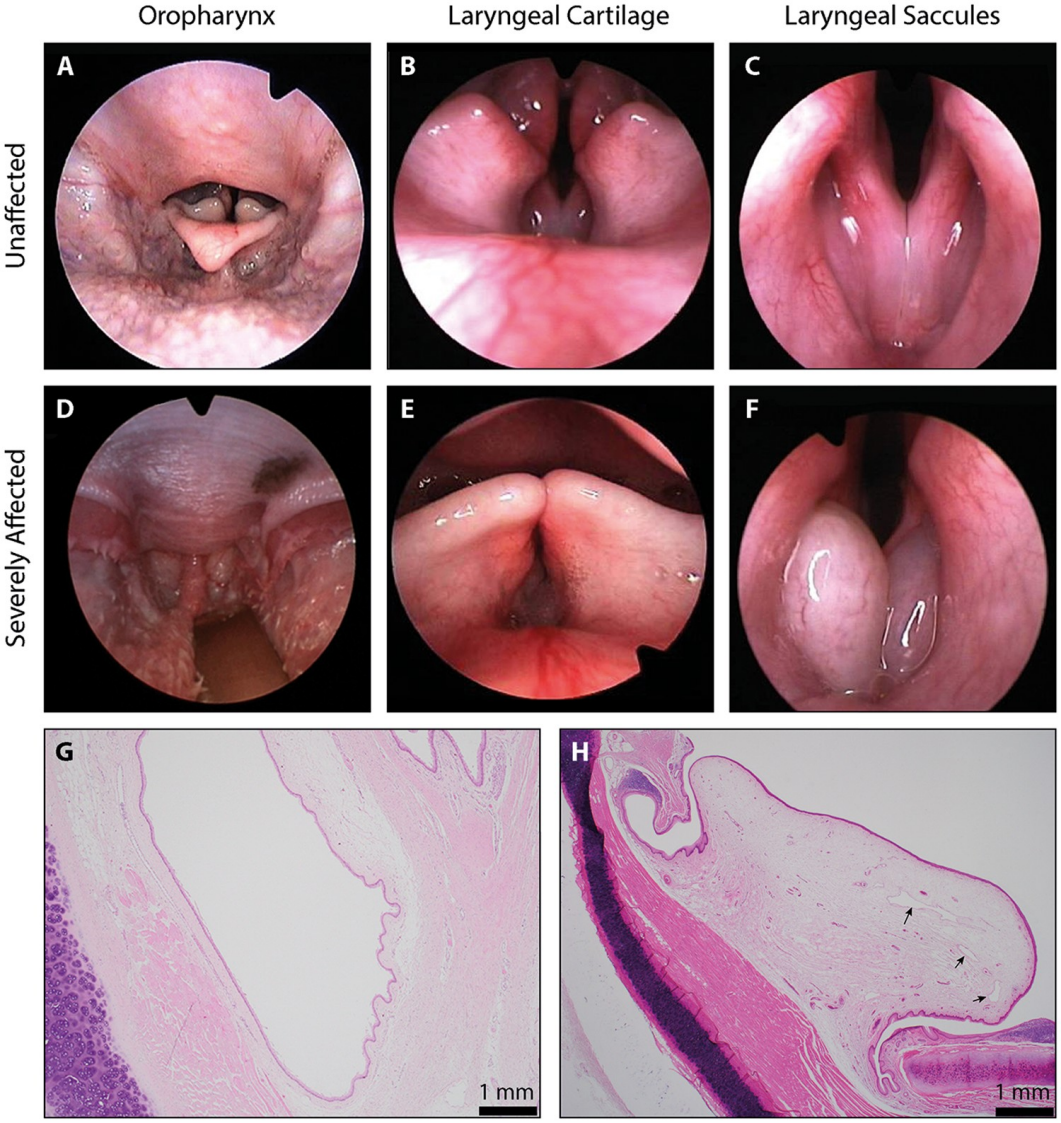
Pathological assessment of Norwich Terrier UAS. (A-F) Images from the laryngoscopic videos that were used to grade anatomical components of the upper airway. Examples graded as ‘normal’ and ‘severely affected’ have been selected for each of the soft palate length (seen in the oropharynx), laryngeal cartilage position and laryngeal saccules. Histological preparations of the laryngeal ventricles from a (G) ‘normal’ and (H) ‘severely affected’ Norwich Terrier. Sections were made perpendicular to the ventricle, at the entrance to the larynx. Arrows indicate dilated lymphatic vessels.

In 2000, an upper airway screening programme for Norwich Terriers was established at the Vetsuisse Faculty of the University of Bern in Switzerland. The programme uses laryngoscopic videos to score ten components of the airway as normal, mild, moderate and severe (S1 and S2 Movies and Table 1) [22]. An overall grade was given for the upper airway condition by combining the ten individual scores. Images taken from the laryngoscopic videos give examples of the soft palate length, laryngeal cartilage and laryngeal saccules graded as ‘normal’ (Fig 2A–2C). In ‘severe’ graded examples, the soft palate is elongated and protruding caudally into the epiglottis (Fig 2D), laryngeal cartilage is inverted into the lumen of the airway (Fig 2E) and the laryngeal saccules are everted (Fig 2F). Histology from an unaffected Norwich Terrier reveals unremarkable connective tissue surrounding the laryngeal saccule (Fig 2G) whilst there is severe oedema and dilated lymphatic vessels in the connective tissue surrounding the laryngeal saccules of a ‘severely’ affected Norwich Terrier (Fig 2H).

**Table 1:** Norwich Terrier phenotype scores. A study population of two-hundred and thirty-three Norwich Terriers representing phenotypic extremes were selected. The number of each airway component graded as ‘normal’, ‘mild’, ‘moderate’ or ‘severe’ are given with the percentage across phenotype in brackets.

Upper Airway Phenotype	Normal	Mild	Moderate	Severe
Laryngeal Saccule Score	11 (4.7%)	75 (32.2%)	66 (28.3%)	81 (34.8%)
Soft Palate Length	43 (18.5%)	67 (28.8%)	90 (38.6%)	33 (14.2%)
Oedema of the Cricoid Mucosa	5 (2.1%)	105 (45.1%)	91 (39.1%)	32 (13.7%)
Oedema of the Pharynx	8 (3.4%)	90 (38.6%)	104 (44.6%)	31 (13.3%)
Oedema of the Oropharynx	41 (17.6%)	128 (54.9%)	56 (24%)	8 (3.4%)
Trachea Shape	119 (51.1%)	87 (37.3%)	21 (9%)	6 (2.6%)
Cartilage Position	97 (41.6%)	92 (39.5%)	39 (16.7%)	5 (2.1%)
Cartilage Stability	125 (53.6%)	76 (32.6%)	30 (12.9%)	2 (0.9%)
Soft Palate Thickness	79 (33.9%)	98 (42.1%)	54 (23.2%)	2 (0.9%)
Cartilage Shape	112 (48.1%)	0 (0%)	121 (51.9%)	0 (0%)

**Fig 3:**
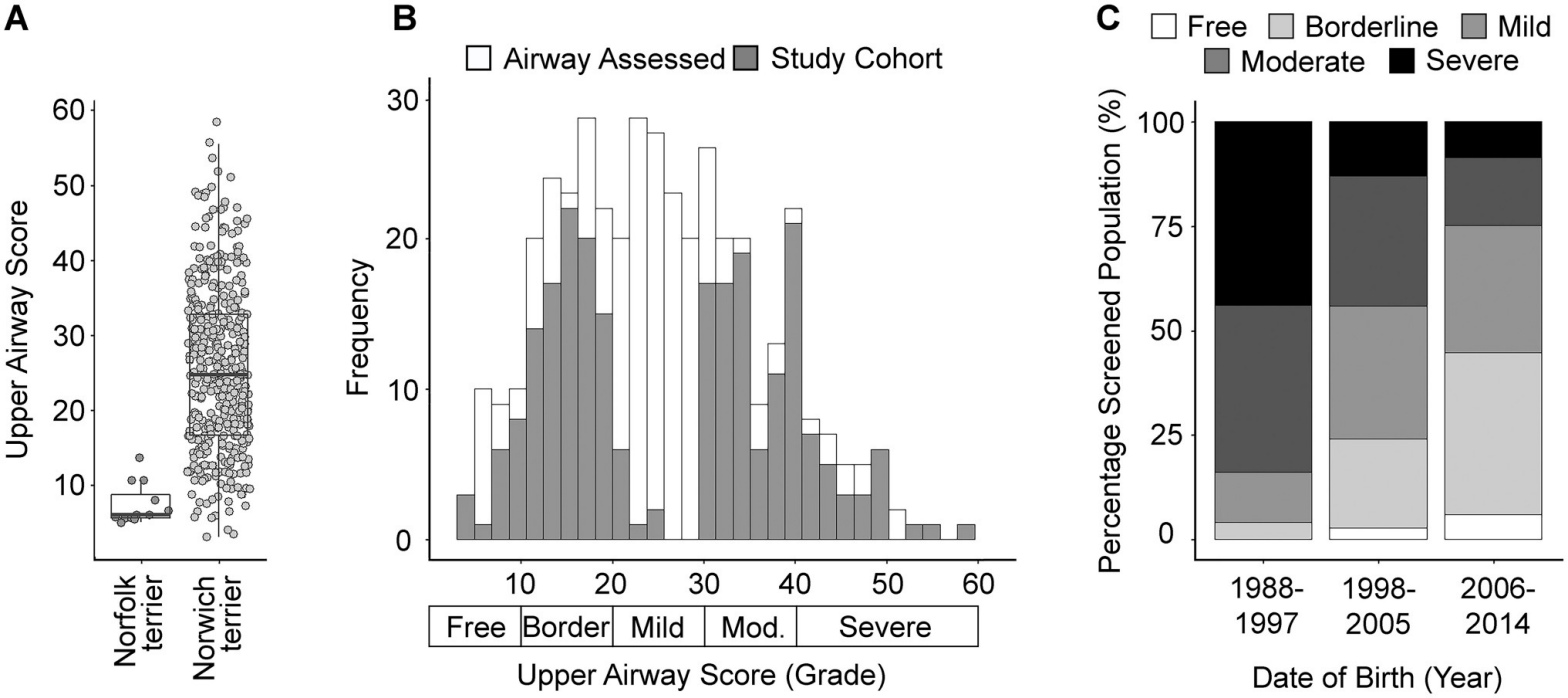
UAS across Norwich Terriers. (A) All Norfolk Terrier upper airway scores were considered clear (n = 12), whilst 277 Norwich Terriers (n = 416) were diagnosed with clinical UAS. (B) Two-hundred and thirty-three Norwich Terriers representing the extremes of those screened made up the study cohort. (C) Breeding recommendations based on upper airway scores have reduced the percentage of severely affected Norwich Terriers over time.

To date, 401 Norwich Terriers in addition to 12 Norfolk Terriers were screened. Two-thirds (65.8%) of Norwich Terriers had overall clinical presentations of UAS ranging from ‘mild’ to ‘severe’ whilst all Norfolk Terriers were unaffected (Fig 3A). We selected 233 Norwich Terriers (109 male, 124 female) representing phenotypic extremes of the phenotypic distribution for our genome-wide association study (GWAS) (Fig 3B). Within this study population, everted laryngeal saccules (81, 35%) and elongated soft palates (33, 14%) were the most common severely-graded phenotype and were only graded as normal in 11 (5%) and 43 (19%) dogs respectively (Table 1). Meanwhile, cartilage stability (125, 54%) and trachea shape (112, 48%) were the most common normal-graded phenotypes. All unique phenotype pairings display a positive Pearson’s correlation coefficient (range: 0.018 to 0.720, median: 0.344) with the exception of the cartilage shape and oedema of the pharynx phenotypes (*r* = -0.116) (S1 Fig). Cartilage position and cartilage stability had the highest correlation of any phenotype pair (*r* = 0.720).

Between 2003 and 2007, The Swiss Terrier Club discouraged breeding dogs exceeding a ‘moderate’ upper airway phenotype. From 2007 onwards, upper airway screening was mandatory for all breeding pairs in Switzerland. As a testament to the coordinated efforts between veterinarians and breeders, the Swiss screening programme observed a reduction in the number of severely affected Norwich Terriers from 44.0% of those born between 1988–1997 to 8.6% for those born in 2006–2014 (Fig 3C). The success of the screening programme underscores the heritability of UAS and suggests that the disease indeed segregates within this population. With cases of UAS reported across continents, the need to develop portable, cost-effective screening strategies became imperative. In order to further reduce the disease prevalence across the Norwich Terrier population and to provide insights into the pathophysiology of respiratory diseases that affect the upper airways of dogs, we sought to establish the genetic underpinnings of the condition.

### Genome-wide association analysis

**Fig 4:**
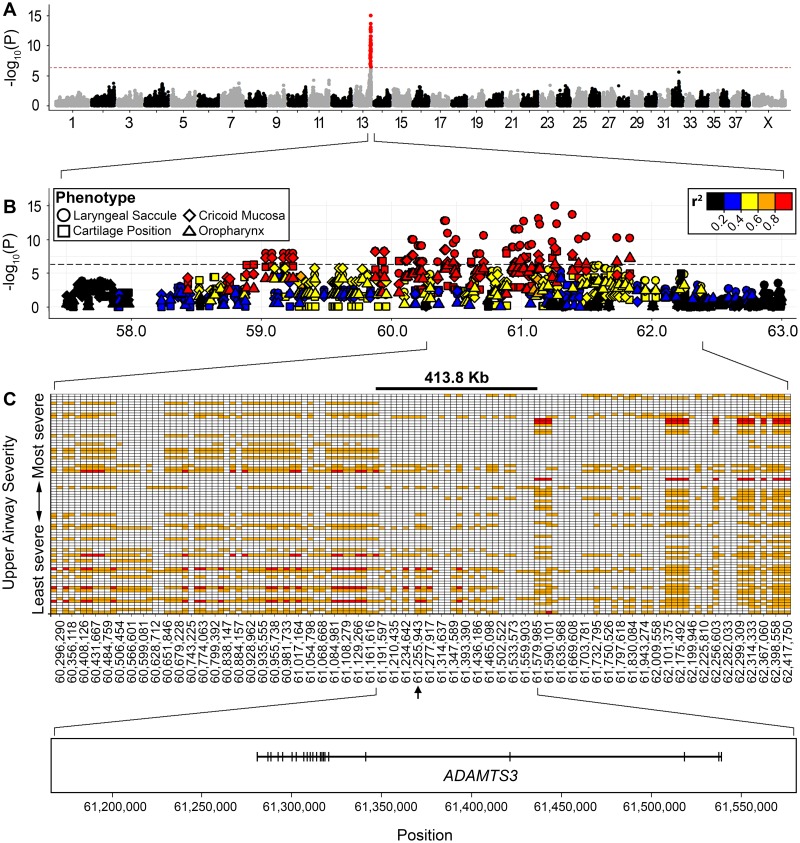
Refinement of CFA13 critical interval. (A) The CFA13 QTL was identified across four upper airway phenotypes including laryngeal saccule score. (B) Marker associations for all phenotypes which returned at least one association surpassing the Bonferroni correction threshold (4.75 x 10^−7^) are overlaid. Point shapes represent phenotypes whilst colour indicates the degree of LD (r^2^) with significant markers. (C) Genotypes were phased for individual Norwich Terriers (horizontal bars) and ordered by the upper airway phenotypes. Only severely affected dogs are shown. Alleles are coloured white, orange and red for homozygous consensus for the risk allele, heterozygous and homozygous alternate alleles, respectively. A 413.8 kb critical interval was defined by at least 3 meiotic recombination events. The index marker (chr13:61,255,943) is identified by an arrowhead. The critical interval encompasses the *ADAMTS3* gene.

Genome-wide association analyses (GWAS) were performed for each of the ten upper airway phenotypes. Four phenotypes including eversion of the laryngeal saccule, oedema of the cricoid mucosa, oedema of the oropharynx and cartilage position returned markers with genome-wide significance (Fig 4A, S2 Fig). The threshold for genome-wide significance was established by Bonferroni correction (-log_10_ [0.05/105,130] = 6.32). Regardless of phenotype, all association tests highlighted the same ~2.9 Mb quantitative trait locus (QTL) spanning 58,941,974–61,830,084 bp on canine chromosome (CFA) 13 with an index marker (TIGRP2P185081_rs9043975) at 13:61,255,943 (Fig 4B, S1 Table). Markers within this broad QTL display high levels of linkage disequilibrium (LD) (r^2^ > 0.2). Due to the modest correlation between individual traits (S1 Fig), many markers (35/57) are significantly associated with at least two phenotypes (S1 Table). Principal components analysis (PCA) of genotypes did not reveal phenotype-related substructure within the study cohort, adding confidence that the signal on CFA13 was truly associated with the disease (S3 Fig).

### Critical interval refinement

Genotypes extending ~1 Mb in both directions from the genome-wide significant markers on CFA13 were phased. Individual dogs were ranked by their disease severity and critical interval boundaries were defined by three meiotic recombinations. This revealed a 413 kb haplotype spanning chr13:61,166,179–61,579,985 that is shared among most severely affected Norwich Terriers (Fig 4C). The disease-associated haplotype was homozygous in 75.3% (61/81) of severely affected dogs whilst it was homozygous in just 18.4% (28/152) of moderately-to-unaffected dogs (S4 Fig). This critical interval spans the entirety of the ADAM metallopeptidase with thrombospondin type 1 motif 3 (*ADAMTS3*) gene, in addition to ~114 kb and ~41 kb of sequence up and downstream of the gene, respectively. No other protein coding genes were annotated within the critical interval.

### Identifying candidate causal variants

**Fig 5:**
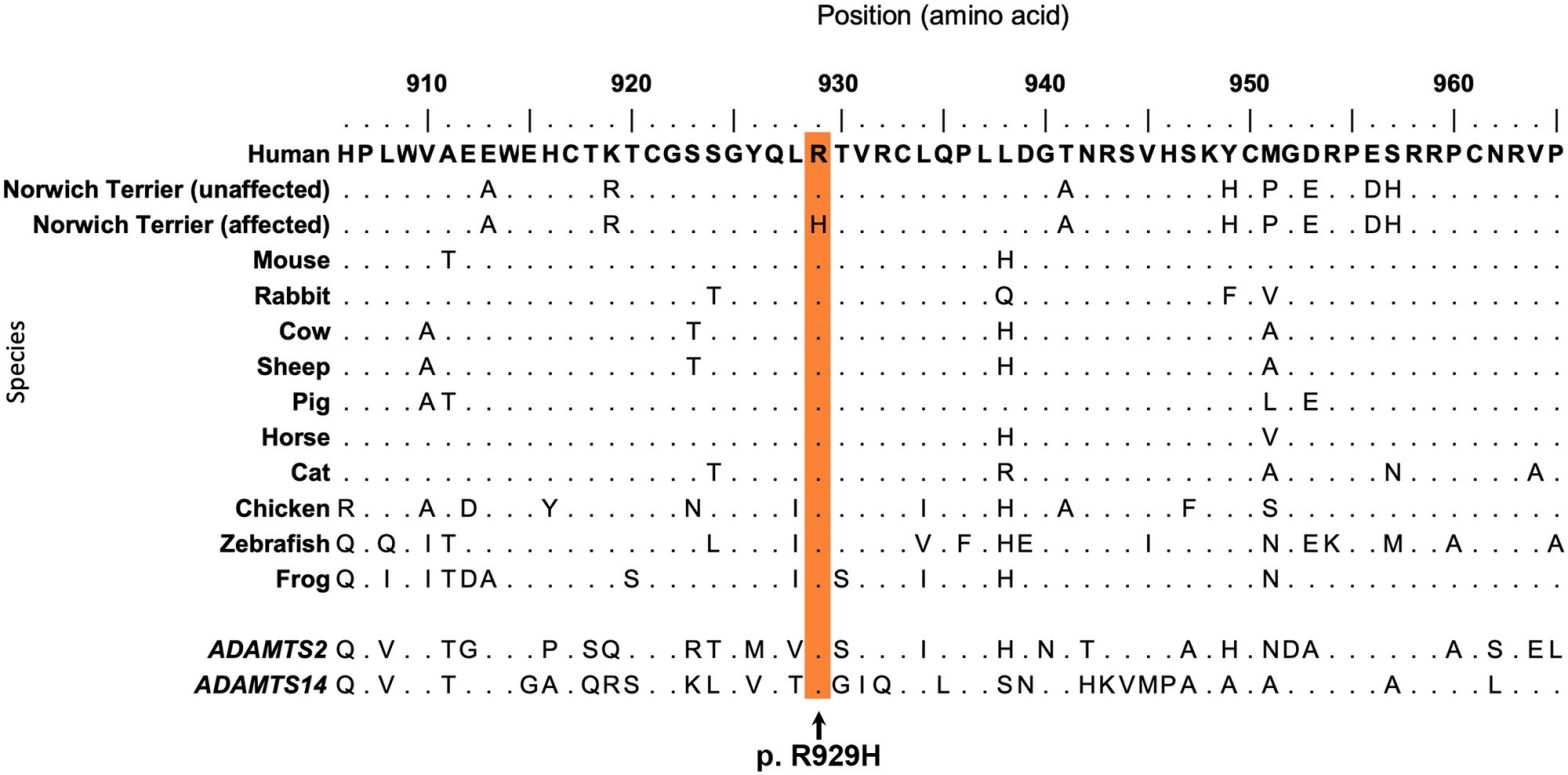
Amino acid conservation across species. Predicted amino acid sequences of the thrombospondin-like domain of vertebrate homologs surrounding the p.R929H missense variant in ADAMTS3 (black arrow). Sequences are conserved across species at the position of the missense mutation in the Norwich Terrier. Paralogs of human ADAMTS3, ADAMTS2 and ADAMTS14 are included.

To search for putative causal variants, we whole genome sequenced four Norwich Terriers representing the extremes of UAS phenotypes. This included two dogs that were homozygous for the CFA13 risk haplotype–one severely affected by UAS and the second seemingly unaffected. The remaining two dogs did not carry the CFA13 risk haplotype and were clinically unaffected. A total of 2,276 variants were called within the 413,806 bp critical interval and subsequently filtered (see Methods), however no variants (SNVs or indels) were compelling candidates for causality based on location and/or interspecies conservation (Table 2, S2 Table). Following visual inspection of the whole genome sequences alongside aligned RNAseq data from a previous study [18], we observed a gap in short-read coverage across all DNAseq and RNAseq datasets at exon 20 of *ADAMTS3* suggesting an error in the CanFam3.1 assembly (S5A Fig). We elected to generate a new local assembly for the CFA13 critical interval using long-read sequencing (see Methods). DNA- and RNA-seq short reads were aligned to the new consensus sequence and revealed that exon 20 of *ADAMTS3* extended an additional 133 bases beyond what was present in CanFam3.1 (S5B Fig). Subsequent variant calling of the new 413,020 bp critical interval identified 1,834 variants. Variants were filtered based on allelic segregation between the disease-associated and alternate haplotypes, leaving a total of 80 single nucleotide variants (SNVs) and small indels. All remaining variants are in complete LD (r^2^ = 1). Two of the remaining variants are exonic–a synonymous variant in exon 21 and a missense variant in the newly defined exon 20 (c.2786G>A) (S2 Table). The missense variant is predicted to change an amino acid of *ADAMTS3* from an arginine to histidine, p.(Arg929His). This arginine is positioned within a thrombospondin type 1 repeat (TSR1) domain and is invariable across mammalian species and close gene paralogs, suggesting evolutionary constraint (Fig 5). Accordingly, a substitution at this position is predicted to be “probably damaging” and “not tolerated” by PolyPhen-2 and SIFT, respectively [25,26].

**Table 2:** Variant calling. Summary of the variants called across the four resequenced Norwich Terriers using Platypus within the critical intervals of the (A) CanFam3.1 and (B) newly created consensus sequence. Variants are filtered by the presence/absence of the risk haplotype.

	CanFam3.1	New Consensus
**Interval (bp)**	61,166,179–61,579,985	1,164,985–1,578,005
**Size (bp)**	413,806	413,020
**Called**	2,276	1,834
**Passed Filters**	231	80
**Intronic**	234	78
**Exonic**	1	2
**Protein Changing**	0	1

### Genotype-phenotype association

**Fig 6:**
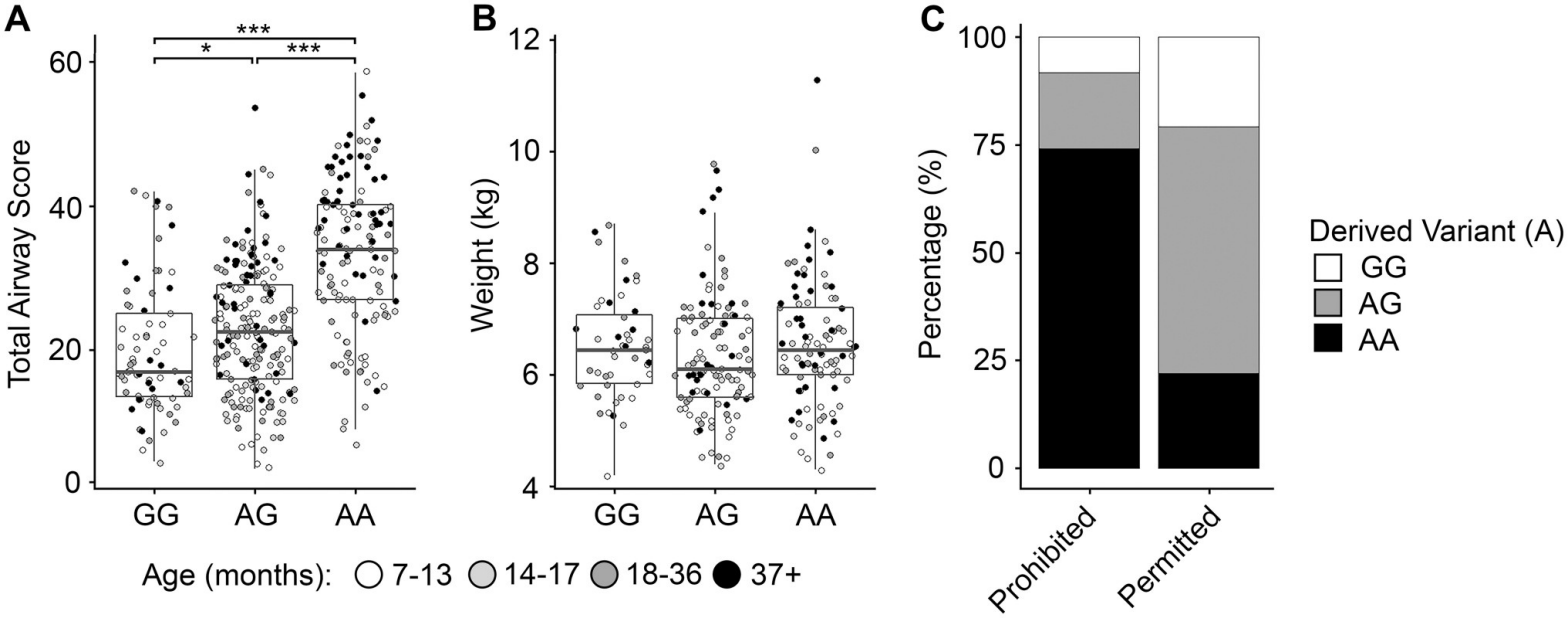
Genotype-phenotype correlation of the *ADAMTS3*:c.2786G>A missense variant. Distributions of (A) total airway scores and (n = 401) (B) weight (n = 250) for the *ADAMTS3* c.2786G>A allele across screened Norwich Terriers. Individuals are coloured by age quartile at the time of upper airway screening. (C) Under the Swiss Terrier Club guidelines, 78% of the Norwich Terriers screened would be permitted to breed whilst 22% would be prohibited. The *ADAMTS3* c.2786G>A allele within these groups are given as a percentage.

We genotyped all Norwich Terriers and Norfolk Terriers screened in the study for the c.2789G>A variant and did not observe the allele among the Norfolk Terrier population (n = 12), as expected, since UAS was not diagnosed in dogs of this breed. However, the risk allele was homozygous in 132 (32.9%) and heterozygous in 195 (48.6%) individuals from the Norwich Terrier cohort (n = 401). Dogs homozygous for the c.2786G>A allele had a significantly greater total upper airway score than those heterozygous (p = 9.10 x 10^−17^) or homozygous (p = 3.08 x 10^−20^) for the ancestral allele (Fig 6A). Seventeen Norwich Terriers were seemingly unaffected by UAS, two of which were homozygous for the c.2786G>A risk allele and nine were heterozygous (Fig 6A). Of note, many of the unaffected Norwich Terriers were young (range 11 to 39, median: 14 months old) at the time they were screened. In contrast, the *ADAMTS3* genotype does not segregate with weight, a suspected respiratory disease risk factor (Fig 6B). Interestingly, by applying the Swiss Norwich Terrier club breeding guidelines to all 401 screened Norwich Terriers, 74.1% of those prevented from breeding are homozygous for the variant, whereas only 22.0% of Norwich Terriers permitted to breed have this genotype (Fig 6C).

Over 1,300 dogs representing up to 114 diverse breeds including representatives of brachycephalic breeds diagnosed with BOAS were screened for the c.2789G>A variant (S3 Table). The disease allele frequency (AF) was observed in the Norwich Terrier (AF = 0.57, n = 401), Bulldog (AF = 0.85, n = 41), French Bulldog (AF = 0.12, n = 23), Staffordshire Bull Terrier (AF = 0.125, n = 8), German Spitz (Mittel) (AF = 0.06, n = 8) and Pomeranian (AF = 0.06, n = 8) suggesting the variant may influence BOAS in the French and English Bulldogs. The ^929^His allele frequency in the human Exome Aggregation Consortium (ExAC) is less than 0.000017 [27].

## Discussion

The incidence of UAS amongst the Norwich Terrier population presented a unique opportunity to identify disease modifiers that may be shared across brachycephalic and non-brachycephalic breeds alike. Leveraging laryngoscopic phenotyping, we identified and refined a QTL to an interval that encompasses a single positional candidate gene, *ADAMTS3*. Following the correction of a local error in the canine reference sequence, we identified an *ADAMTS3* c.2786G>A missense variant that is associated with cases of UAS in the Norwich Terrier. Subsequently the French Bulldog and Bulldog, breeds susceptible to brachycephalic obstructive airway syndrome, were also identified as carriers of the *ADAMTS3* missense allele.

The ADAMTS proteins are a large family of protease enzymes [28,29]. The procollagen N-proteinases, which includes *ADAMTS3*, are a subgroup of this family which were first shown to be expressed in cartilage amongst other tissues [30–32]. Within cartilage, *ADAMTS3* has a substrate specificity for procollagen type II, which it cleaves to stimulate the maturation into collagen II, the major isoform of cartilage [33–37]. *ADAMTS3* also has an important signalling function, as it proteolytically activates vascular endothelial growth factor-C (VEGF-C), which in turn promotes lymphangiogenesis [38,39]. Loss of this signalling function in humans causes Hennekam lymphangiectasia-lymphedema syndrome 3, a condition characterised by lymphedema and distinct facial features including hypertelorism and a flat nasal bridge [40–42]. This oedematous human phenotype is recapitulated in two different *Adamts3* knockout mouse lines which were reported to have severe defects in lymphatic development [43–45]. Both knockout lines resulted in perinatal lethality with Ogino *et al*., reporting death was due to apparent breathing problems. Interestingly, this line also presented with abnormal rib development and significantly rostrocaudally shortened skulls [43]. Whilst these studies did not specifically examine the tissues of the upper airways, the oedematous phenotype draws parallels with our observations in affected Norwich Terriers which carry the *ADAMTS3* c.2786G>A variant.

Interestingly, in both the *ADAMTS3* knock out mouse and cases of human Hennekam lymphangiectasia-lymphedema, craniofacial abnormalities are reported in conjunction with aberrant lymphatic development. Based on our analysis of rostra, we did not detect morphological differences between affected and unaffected Norwich Terriers, nor Norfolk Terriers. Similarly, there were no distinguishable differences in height or weight between the disease groups. Given the limitations of these assessments (e.g. limited skull scans, imprecise postcranial measurements), it is possible that morphological differences between disease groups were undetected.

Traditionally, the brachycephalic skull conformation has been considered the major predisposing factor to airway obstruction in brachycephalic breeds such as the (English) Bulldog and French Bulldog. The parallels in the upper airway oedematous phenotype in both brachycephalic dogs and the Norwich Terrier, along with the high prevalence of the *ADAMTS3* c.2786G>A variant in brachycephalic breeds raises the possibility that it promotes airway disease in these other breeds. This presents a new paradigm in our understanding of obstructive airway disease in that both a compromising skull conformation and a predisposition to oedema of the airway contributes to disease presentation. Complex genetic effects, which may include *ADAMTS3* c.2786G>A could explain the varying susceptibility to BOAS across brachycephalic breeds.

The *ADAMTS3* c.2786G>A variant could not be separated from a further seventy-nine SNVs and small indels during filtering due to the long-range LD [46,47]. However, none of the additional intronic variants were compelling based on their position of cross-species conservation. Conversely, the arginine 929 residue in *ADAMTS3* is highly conserved across orthologs and its close paralogs, *ADAMTS2* and *ADAMTS14*, from diverse vertebrates. Arginine 929 is located in the third of four thrombospondin type 1 repeats (TSR1) within *ADAMTS3*. The TSR1 repeats are thought to contribute to substrate binding and interactions with the extracellular matrix [48,49]. Although the variant site is outside the catalytically active metalloproteinase domain of *ADAMTS3*, the canine Arg929His substitution might change or even disrupt the correct folding of the third TSR1 repeat. It has been shown that several arginine residues within the TSR1 domain contribute to the so-called central arginine layer, an important element of the three-dimensional structure of TSR1 repeats [50]. Thus, it is plausible that p.Arg929His might alter the functional properties of *ADAMTS3*. The exact functional impact of the Arg929His substitution requires further investigation.

The identification of *ADAMTS3* in obstructive airway syndrome across dogs suggests a likely new role for the gene in effective respiratory function. This discovery warrants further longitudinal studies to assess possible correlations between the risk allele and complications during corrective upper airway surgery, where oedema of the upper airway can predispose dogs to post-operative complications. Identification of the *ADAMTS3* c.2786G>A risk allele is a critically important step to understanding the aetiology of airway disease, which at present is poorly understood. Future studies are warranted to understand the potential of the c.2786G>A allele’s potential use as a diagnostic marker of disease.

## Methods

### Ethics statement

All animal experiments were performed according to the local regulations. The dogs in this study were examined with the consent of their owners. The study was approved by the Federal Food Safety and Veterinary Office at the Federal Department of Home Affairs, Switzerland (registration number 2.03.03). Swiss biobanking was approved by the “Cantonal Committee For Animal Experiments” (Canton of Bern; permits 22/07, 23/10, and 75/16) and the R(D)SVS Veterinary Ethical Review Committee (20 16, University of Edinburgh).

### Participants and upper airway assessment

All animal experiments were performed according to the local regulations. The dogs in this study were examined with the consent of their owners. The study was approved by the “Cantonal Committee For Animal Experiments” (Canton of Bern; permits 22/07, 23/10, and 75/16) and the R(D)SVS Veterinary Ethical Review Committee (20 16, University of Edinburgh). A full description of the upper assessment was described previously [22]. In short, the upper respiratory tracts of 401 Norwich Terriers and 12 Norfolk Terriers were assessed *in situ* during endoscopic examination and scored retrospectively from video footage. All evaluations were conducted by a single veterinary surgeon. Subsequently, each of the ten phenotypic components of the airway (soft palate length, soft plate thickness, laryngeal saccule, cartilage shape, cartilage stability, cartilage position, oedema of the oropharynx, oedema of the pharynx, oedema of the cricoid mucosa and shape of the trachea) were scored on a range from 1 to 4 representing ‘normal’, ‘mild’, ‘moderate’ and ‘severe’ respectively by authors PS, ED and UR. A custom R script was used to generate Pearson’s correlation and dendrogram. Individual phenotype scores were weighted and summed to give the total airway score.

Two-hundred and thirty-three Norwich Terriers (109 male, 124 female) representing the extremes of upper airway phenotypes formed the study cohort. Participants varied in age from 7 to 188 months (median = 18 months).

### Histology

H&E stains were done as previously described from dogs donated posthumously [51].

### Skull shape sssessment

The geometric morphometric analysis of 3D skull reconstructions generated from computer tomography scans have been described previously [18]. Linear measurements of the hard palate were made, and the influence of allometry regressed using the neurocranium centroid size. PCA of the viscerocranium of 565 dogs representing 96 UK Kennel Club registered breeds permitted the comparison of face shapes.

### Genotyping and genomic analysis

Whole blood samples were taken and stored in EDTA at 4°C prior to gDNA extraction following the whole blood protocol of the Nucleon BACC Genomic DNA Extraction Kit (RPN-8502, GE Healthcare Life Sciences). Genotypes were generated using the Illumina 170,000 SNV CanineHD bead chip by Edinburgh Genomics, UK and mapped to the CanFam3.1 coordinates.

SNVs with minor allele frequencies < 0.05 and individuals with > 0.1 missing genotypes were removed using PLINK (v1.90) [52]. Genotypes were imputed using a two-step process that included pre-phasing by SHAPEIT [53] and imputation by IMPUTE2 [54]. A total of 105,130 SNVs were used by GEMMA (v0.94.1) in a univariate linear mixed model to perform GWAS [55]. A kinship matrix was implemented during the analysis with age and sex used as covariates. A Bonferroni correction threshold was used to determine statistically significant SNVs (-log_10_ [0.05/105,130] = 6.32). The LD of significant SNVs with all other markers in a 50 variant window was calculated using the independent pairwise test in PLINK (v1.90).

Phased haplotypes encompassing the index SNV (TIGRP2P185081_rs9043975) at chr13: 61,255,943 were ordered by UAS severity. The order was dictated by the four phenotypes returning significantly associated index SNVs (laryngeal saccule > cartilage position > oedema of the cricoid mucosa > oedema of the oropharynx) such that dogs with the most severe grade of all four phenotypes were positioned at the top. A consensus risk haplotype was the most frequent haplotype within the most severe scoring dogs, appearing in 98 of 162 chromosomes from severely affected dogs. Risk alleles were coloured based on whether they matched this consensus haplotype. The critical interval boundaries were defined by three or more meiotic recombination events across the severely affected Norwich Terriers.

### Sequencing and variant analysis

Four Norwich Terriers representing upper airway phenotypic extremes were whole genome sequenced to an average coverage of 15.9x. Two Norwich Terriers were homozygous for the disease-associated haplotype with one severely affected and the second apparently unaffected. The remaining two dogs did not have the disease-associated haplotype and were unaffected. DNA libraries were prepared using the TruSeq DNA PCR-free Library Preparation Kit. The Illumina HiSeq 2500 system sequenced 125 bp paired-end libraries with and average insert size of 419 bp. Reads from each resequenced Norwich Terrier were aligned to CanFam3.1 assembly using BWA-MEM [56] and variants within the critical interval chr13:61,166,179–61,579,985 were called for the CanFam3.1 and Zoey2.3 assembly using Platypus (v0.8.1) [57]. Two Norwich Terriers homozygous for the disease-associated haplotype with differing phenotypes were selected with the potential of discovering the ancestral haplotype prior to the introduction of the causal variant(s). To this end, for positions that had calls for all four Norwich Terriers, filtering criteria required variant(s) to be homozygous and exclusive to the affected dog, however this returned no variants. We hypothesised that the unaffected dog homozygous for the disease-associated haplotype was still subclinical due to the age of scoping at 1.2 years. Subsequently, filtering criteria required variants to be homozygous derived in the disease-haplotype carrying dogs and homozygous ancestral in those not carrying it.

Norwich Terrier DNA and CanFam3.1-aligned RNAseq reads were viewed in IGV [58] which revealed an error in the reference sequence [18]. To resolve this error, we compared the local assembly of a Great Dane produced from PacBio long reads. In addition, we generated a *de novo* assembly from three bacterial artificial chromosomes (BACs) originally used for the CanFam3.1 assembly. BACs spanning the critical interval were sourced from the BACPAC Resource Center, Children’s Hospital Oakland Research Institute, California, USA (CH82-24F19, CH82-379O18 and CH82-101M10). Following BAC isolation (PhasePrep DNA Kit, Sigma-Aldrich, NA0100), a DNA library was prepared from an equimolar mix of the three BACs for a single 1D barcode-free gDNA sequencing run using the Oxford Nanopore Technologies MinION platform (SQK-LSK109, R9.4). A pipeline including Albacore (v2.0.1), Canu (v1.5) and Nanopolish (v0.8.4) was used to base call, construct contigs and improve consensus sequence respectively. The consensus sequences of both long-read platforms were in agreement and resolved the error underlying exon 20 of *ADAMTS3*, though neither platform’s base calling resolved a ~40 bp intronic stretch of guanines downstream of exon 20.

Norwich Terrier short-read data (European Nucleotide Archive study accession PRJEB16012) and RNAseq data (European Nucleotide Archive study accession PRJEB17926) from a previous study were realigned to the new consensus using BWA-MEM and STAR (V2.5.1) respectively with default parameters [18,59]. The RNAseq data was used solely to confirm exonic structure whilst the DNAseq data was used to repeat variant calling as previously described.

Protein sequences of *ADAMTS3* homologs (HGNC:219) across species were downloaded from Ensembl and aligned using a ClustalW multiple alignment [60]. XP_539311, a low-quality protein prediction of canine *ADAMTS3* differed substantially from other species in its sequence corresponding to exon 20 –likely due to 133 bp of exon 20 missing from the CanFam3.1 assembly. To this end, the predicted amino acid sequence for the Norwich Terrier was created using comparative RNAseq alignment from nine dogs, representing eight breeds which were in full agreement for exon structure [18]. Residue positions are relative to the start codon. The thrombospondin-like domain (PS50092) was predicted using PROSITE database of protein domains [61].

To genotype the *ADAMTS3*:c.2786G>A variant, forward (ACACACGAACCCAGGCACAC) and reverse (GGCCTGGGAGCACTGCAC) primers were designed to amplify the region. PCR products were Sanger sequenced by Edinburgh Genomics, UK. All breeds used for genotyping were owner-reported.

## Supporting information

S1 Fig Phenotype correlations. Pearson’s correlation scores between upper airway phenotypes with dendrogram indicating relationships between phenotypes. Phenotypes returning significant associations in the GWAS are marked with (*).
(TIF)

S2 Fig All GWAS returning significant associations. Manhattan plots for (A) cartilage position, (B) laryngeal saccule, (C) oedema of the cricoid mucosa and (D) oedema of the oropharynx. The red dashed line denotes Bonferroni correction threshold (4.75 x 10^−7^) and maximum significance values for index SNPs of each test are given.
(TIF)

S3 Fig Array genotype PCA. Principal component 1 and 2 of the array genotypes do not segregate by laryngeal saccule score.
(TIF)

S4 Fig Complete haplotypes. Phased haplotypes (horizontal rows) for the region surrounding the index marker (chr13:61,255,943; arrowhead) on CFA13 for all 233 Norwich Terriers in the study cohort. Individuals are ranked by phenotype severity in order of their GWAS significance (laryngeal saccule > cartilage position > oedema of the cricoid mucosa > oedema of the oropharynx).
(TIF)

S5 Fig Error in CanFam3.1 Scaffold. DNA and RNA short-read sequencing data aligned to the (A) CanFam3.1 and (B) newly created consensus sequence for the region. Read coverage across the 5’ end of exon 20 and its immediate intron is lost. The error is corrected in the new consensus as indicated by complete coverage in the region. Black arrow indicates the position of the *ADAMTS3* c.2786G>A variant.
(EPS)

S1 Table Significant SNVs. Four phenotypes have markers that surpass a Bonferroni correction threshold which correspond to the same QTL on CFA13. Only *p*-values exceeding this threshold (4.76 x10^-7^) are reported. Alleles are reported as ancestral > derived.
(XLSX)

S2 Table List of remaining variants. Variants passing filtering criteria when aligned to the CanFam3.1 and new consensus (Zoey2.3) assemblies.
(XLSX)

S3 Table Multiple breeds genotyped for *ADAMTS3* variant. ADAMTS3:c.2786G>A genotypes of 1,353 dogs from up to 114 different breeds. The total number of dogs genotyped per breed (n) is given and the frequency of genotypes within breeds is given in brackets as a percentage.
(XLSX)

S1 Movie Laryngoscopic video of a normal Norwich Terrier airway. An example laryngoscopic video of a Norwich Terrier with largely unremarkable larynx and pharynx. There is slight oedema of the pharyngeal vault and of the cricoid mucosa. The rima glottidis appears wide and open.
(MP4)

S2 Movie Laryngoscopic video of a severely affected Norwich Terrier airway. An example laryngoscopic video of a Norwich Terrier with a severe clinical upper airway phenotype. There is excessive oedema of the oropharynx, pharyngeal vault, cricoid mucosa and mucosa of the laryngeal saccules. The rima glottidis appears to be extremely narrow.
(MP4)
