# Supplementary figures and images for: An ADAMTS3 missense variant is associated with Norwich Terrier upper airway syndrome
Source: PLoS Genet. 2019 May 16;15(5):e1008102. doi: 10.1371/journal.pgen.1008102 (PMC6521993; doi:10.1371/journal.pgen.1008102)

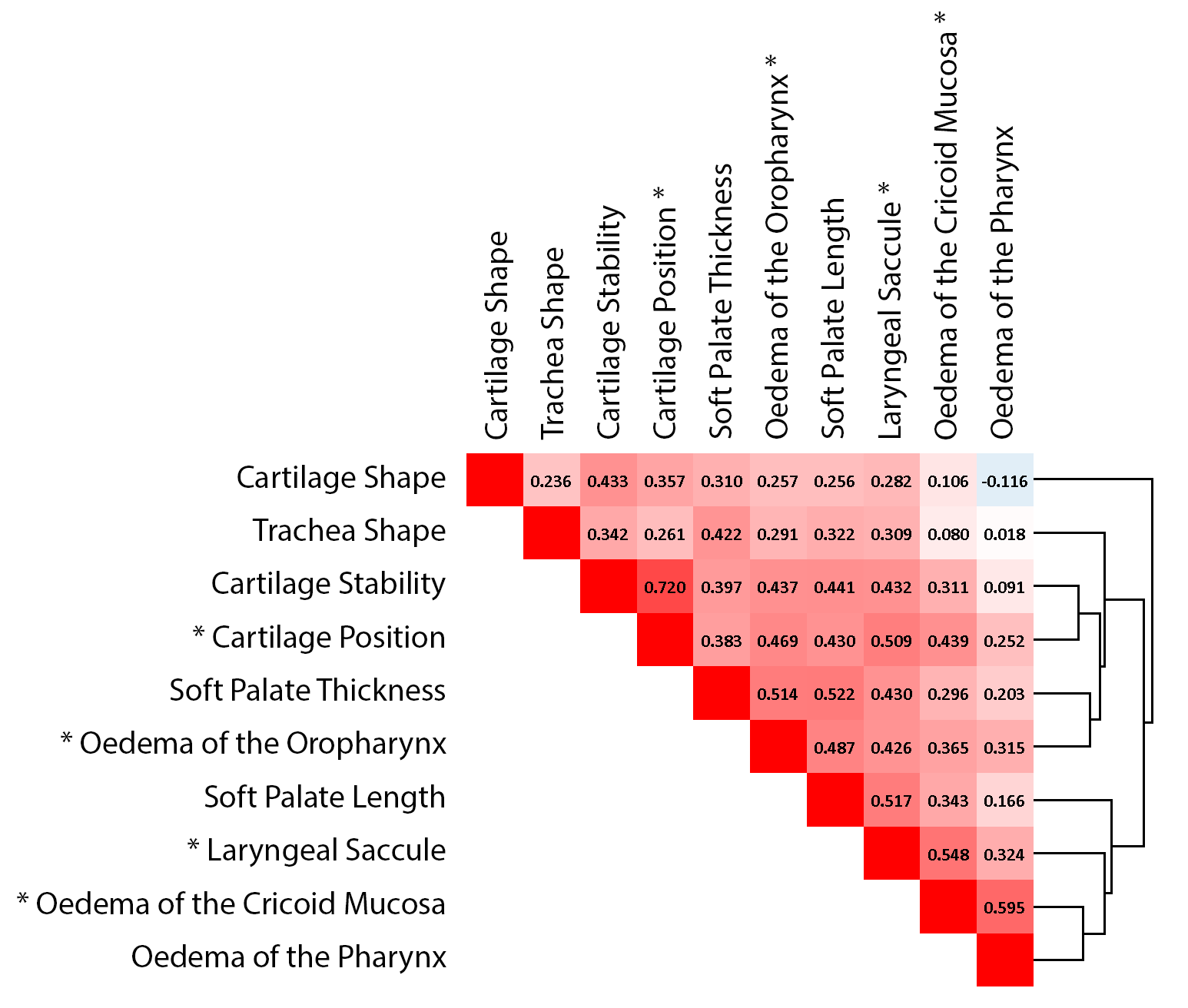

Supplement: S1 Fig — Pearson’s correlation scores between upper airway phenotypes with dendrogram indicating relationships between phenotypes. Phenotypes returning significant associations in the GWAS are marked with (*). (TIF) [file pgen.1008102.s001.tif]

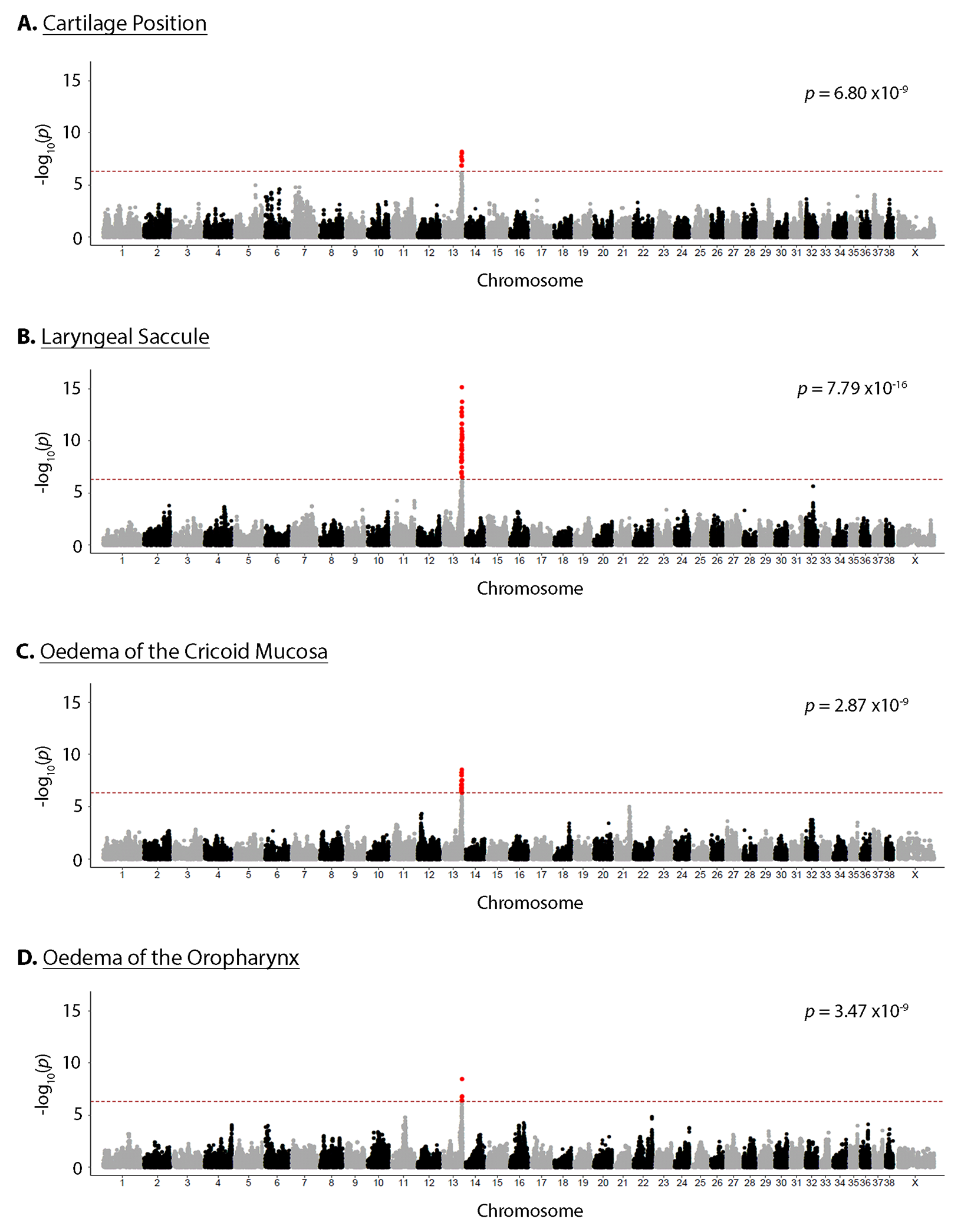

Supplement: S2 Fig — Manhattan plots for (A) cartilage position, (B) laryngeal saccule, (C) oedema of the cricoid mucosa and (D) oedema of the oropharynx. The red dashed line denotes Bonferroni correction threshold (4.75 x 10^−7^) and maximum significance values for index SNPs of each test are given. (TIF) [file pgen.1008102.s002.tif]

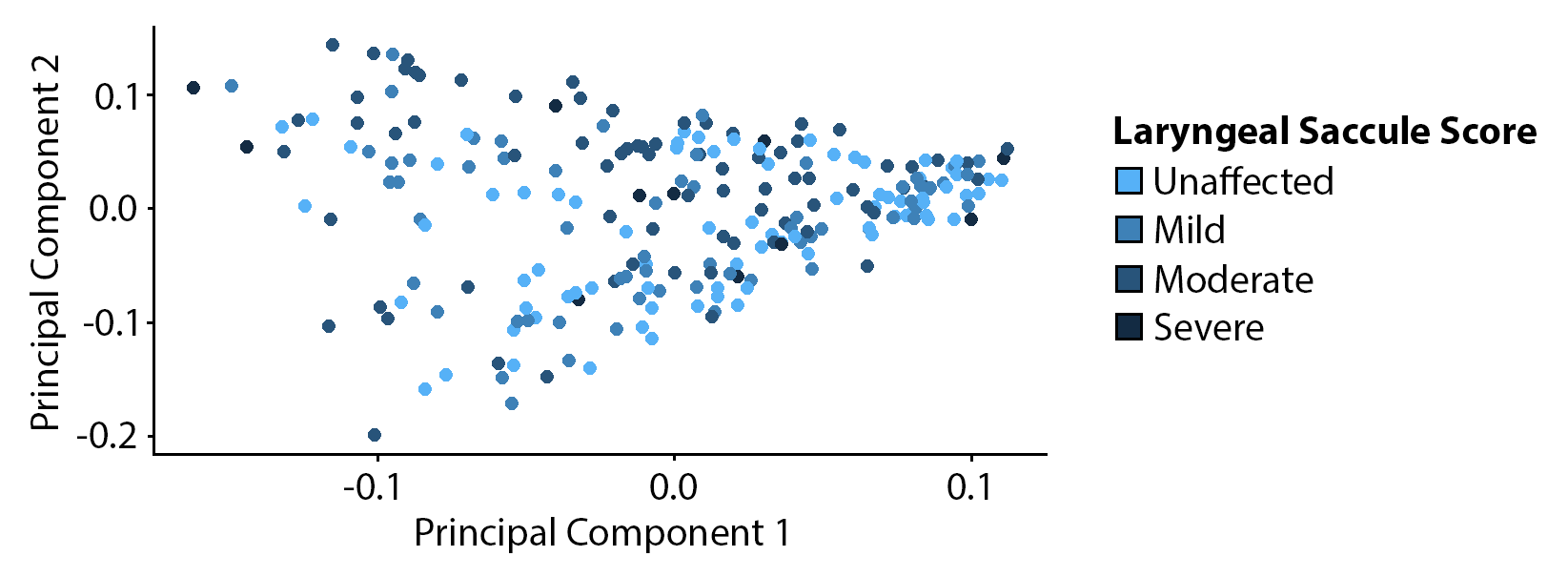

Supplement: S3 Fig — Principal component 1 and 2 of the array genotypes do not segregate by laryngeal saccule score. (TIF) [file pgen.1008102.s003.tif]

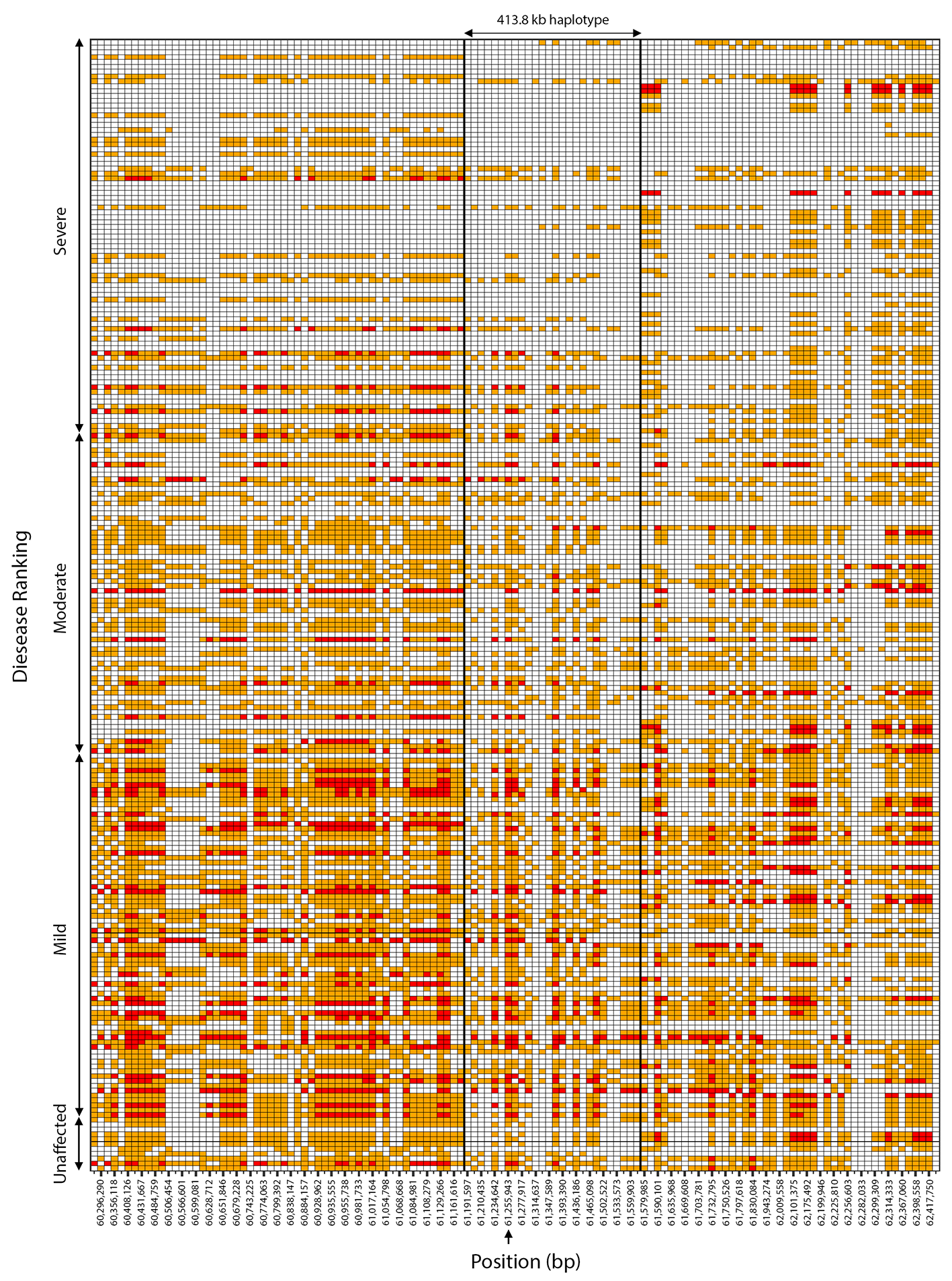

Supplement: S4 Fig — Phased haplotypes (horizontal rows) for the region surrounding the index marker (chr13:61,255,943; arrowhead) on CFA13 for all 233 Norwich Terriers in the study cohort. Individuals are ranked by phenotype severity in order of their GWAS significance (laryngeal saccule > cartilage position > oedema of the cricoid mucosa > oedema of the oropharynx). (TIF) [file pgen.1008102.s004.tif]
